# Deep Vein Thrombosis in Acute Stroke - A Systemic Review of the Literature

**DOI:** 10.7759/cureus.1982

**Published:** 2017-12-23

**Authors:** Muhammad T Khan, Asad Ikram, Omar Saeed, Taha Afridi, Cathy A Sila, Matthew S Smith, Khadija Irshad, Ashfaq Shuaib

**Affiliations:** 1 Neurology, Charleston Area Medical Center; 2 Department of Neurology, University of New Mexico School of Medicine, Albuquerque, New Mexico, USA; 3 Department of Neurology, Zeenat Qureshi Stroke Insitute; 4 Hood College, Hood College, Maryland; 5 Department of Neurology, University Hospitals Case Western Reserve University School of Medicine; 6 Neurology, West Virginia University; 7 Department of Neurology, Henry Ford Health System; 8 Department of Neurology, University of Alberta

**Keywords:** dvt, acute stroke, acute stroke therapy, pneumatic compression devices, management guidelines, treatment, deep vein thrombosis

## Abstract

We present a systemic review of available literature on the complications of deep venous thrombosis that develops in patients presenting with acute stroke. There are several pharmacological and physical treatment options available and used. We aim to summarize the management plans currently used at different centers. In conclusion, low-dose anticoagulant therapy for ischemic stroke is recommended. In the case of intracerebral hemorrhage, pneumatic sequential compression devices should be placed initially, followed by the administration of ultra-fractioned heparin on the next day, and then oral anticoagulant therapy to replace the heparin after a week in high-risk patients. Similar prophylactic treatment recommendations are used for subarachnoid hemorrhage.

## Introduction and background

Deep vein thrombosis (DVT) is a serious complication in stroke patients and may lead to the devastating consequences of a pulmonary embolism. Both pharmacological and physical methods are used to prevent DVT. New insightful research is exploring drugs and laboratory diagnostic parameters that will aid in the early diagnosis and timely management of DVT.

Epidemiology

DVT commonly occurs in the setting of stroke and can be a fatal complication if it leads to pulmonary emboli. In immobilized post-stroke patients, the incidences of DVTs vary from 10-75%, depending on the diagnostic method and time of evaluation [1-2]. Asymptomatic DVT and clinically evident DVT vary in their prevalence, the latter being 2-10% after an acute stroke [3-6]. The onset of development of a DVT after acute stroke can be as early as the second day, peaking between Days 2 and 7; if left untreated, proximal DVT have a 15% risk of death [7]. Venous thromboembolism (VTE) is also very commonly seen in patients with subarachnoid hemorrhage (SAH) or an intracerebral hemorrhage (ICH). The risk of DVT/pulmonary embolism (PE) may even be higher in patients with SAH and ICH but smallest with the transient ischemic attack (TIA) [8].

The most common cause of mortality from a DVT after a stroke is a PE, accounting for 13-25% of early deaths, and their incidence usually ranges from 1-3% in the first few months after a stroke [3-5].

The risk factors for DVT in acute stroke are advanced age, high National Institute of Health Stroke Scale (NIHSS) score, hemiparesis, immobility, female gender, atrial fibrillation, receipt of intravenous or intra-arterial tissue plasminogen activator (TPA), and admission to an academic hospital [9-11]. A recent large prospective study based on a cohort of 30,000 members of a general population in Norway documented the incidence of acute stroke and the risk of DVT and PE in patients who had a stroke. The study found that the risk of DVT and PE in their group of participants with acute ischemic stroke was independent of traditional cardiovascular risk factors, implying that stroke and related conditions (mainly immobility) are the main contributors to the VTE risk [12].

The initial test of choice for diagnosing peripheral venous thrombosis is ultrasound due to its accuracy, low cost, portability, and safety [13]. In addition, Doppler techniques provide direct information regarding flow physiology [14-16]. Either computed tomography (CT) or magnetic resonance imaging (MRI) can be used, particularly when studying central veins. Other screening tests employed are 125-I fibrinogen scanning and contrast venography. A 16-point early clinical prediction scale has been suggested to identify patients at high risk for DVT after an ischemic stroke but needs further study and validation [17].

New DVTs increased the risk of three-month mortality significantly with no influence on the combined risk of death and dependency. Increased serum C-reactive protein (CRP) levels, alongside a normal fibrinogen level, can predict the development of a new DVT. Such patients may then be reasonably protected with further DVT prophylaxis [1]. Studies that evaluated clinical factors alone could not adequately differentiate between immobile stroke patients at high or low risk, and therefore, did not formulate customized DVT prophylaxis plans. In the future, research should investigate factors, including simple blood tests, which can stratify patients according to the risk of VTE and also the risk of bleeding and identify which of these patients would have a net benefit from pharmacological prophylaxis [15]. In patients with hemorrhagic stroke, early anticoagulation is associated with a significant reduction in PE, a non-significant reduction in DVT or death, and a non-significant increase in hematoma enlargement [18].

## Review

Materials and methods:

We conducted a systematic review of all published studies until June 2017 examining DVT prophylaxis in acute stroke. All studies were searched in the English language using Medline, Embase, PubMed, and the Cochrane Database. Literature search and screening strategy, studies selection, data extraction methods, and risk assessment of bias were made using predetermined criteria and supervised by one of the senior authors to maintain quality. Data were searched using the terms DVT, prophylaxis, and acute stroke. The database search identified cross-sectional studies, randomized control trials, meta-analysis, review articles, and original articles on adult human subjects.  

All studies were selected which met the eligibility criteria for inclusion. The content was then reviewed by two independent investigators to determine eligibility. Any discrepancies were further resolved by using a group discussion in which a decision was made to include or exclude the study according to previously set criteria. Quality assessment was made by both reviewers evaluating whether there was a clear research question with specific results, clear description of inclusion and exclusion criteria, sound methodology, generalizability, and mentioning of limitations.

Pharmacologic prophylaxis

In this review, we summarize the efficacy and safety of different anticoagulant strategies in the prevention of VTE in patients with acute stroke. In patients with acute ischemic stroke, a reduction of venous thromboembolic events was noted when low-molecular-weight-heparins were used [19]. However, this slightly increased the risk of extracranial bleeding. The combined death and disability rates were reduced with the use of LMWH but could increase complications related to hemorrhage [19]. When compared with standard unfractionated heparin (UFH), the LMWHS or heparinoid appeared to decrease the occurrence of DVTs after acute ischemic strokes [20]. Comparison of UFH and LMWH revealed that low-dose LMWH had a benefit/risk ratio in patients with acute ischemic stroke by decreasing the risk of both DVT and pulmonary embolism without a clear increase in intracranial hemorrhage [21]. Based on these studies, the conclusion was that UFH and LMWH are both effective in reducing DVT and PE in patients with stroke at the cost of a slightly increased risk of hemorrhage (intracerebral and extracranial).

Unstructured Review of Unfractionated Heparin and Low-Molecular-Weight Heparin

a)      Out of a series of randomized controlled trials published through January 2005 involving 23,043 patients reviewed for a comparison of early administration of either LMWH or UFH with control (placebo or no treatment) for VTE prevention in patients with acute ischemic stroke, 16 trials met the inclusion criteria [22]. Low-dose UFH (< 15,000 units/day) showed a decreased risk of DVT with no significant effect on risk of PE and intracranial or extracranial hemorrhage. High-dose UFH (> 15,000 units/day) reduced the risk of PE but subsequently increased the risk of both major intracranial and extracranial hemorrhage. Low-dose LMWH (< 6,000 IU/day) or a weight-adjusted dose of < 86 IU/kg/day reduced the risk of both DVT and PE and showed no increased risk of major intracranial or extracranial hemorrhage. Finally, the high-dose LMWH (> 6,000 IU/day or > 86 IU/kg/day) also decreased the risk of both DVT and PE but increased the risk of both major intracranial and extracranial bleeds [22]. The authors concluded that low-dose LMWH offered the best benefit to risk ratio for VTE prophylaxis. The numbers needed to treat (NNT) with low-dose LMWH to prevent DVT and PE were 7 and 38, respectively [22].

b)      In a controlled trial that assessed UFH versus placebo effects directly and also studied VTE as a primary consequence, 305 elderly patients having an acute stroke were allocated to treatment or control groups randomly [23]. The treatment group showed a significant reduction in DVT compared with placebo as assessed by fibrinogen scanning. In patients who died, the treatment group showed an association with a significant reduction in PE at postmortem examination compared with placebo (10% versus 46%) [23].

c)      The International Stroke Trial (IST) evaluated VTE as a secondary outcome. In this trial, more than 19,000 patients were allocated to subcutaneous unfractionated heparin treatment (12,500 units twice daily or 5,000 units twice daily) versus no heparin and aspirin, 300 mg daily, versus no aspirin [5, 21]. The heparin treatment group showed a decline in the frequency of fatal or nonfatal PE when compared with the group not treated with heparin (0.8% versus 0.5%), but the reduction was significant only in patients also given aspirin, 300 mg daily. Heparin therapy was also associated with a significantly increased risk of intracranial hemorrhage compared with no heparin (1.2% versus 0.3%) in those patients who did not receive aspirin [5].

d)      The open-label prospective randomized evaluation of the “Watchman Left Atrial Appendage Closure Device in Patients with Atrial Fibrillation Versus Long-term Warfarin Therapy” (PREVAIL) trial assessed 1,762 patients with acute ischemic stroke who had leg weakness and could not walk unassisted were randomly assigned to subcutaneous LMWH (enoxaparin, 40 mg daily) or subcutaneous heparin, 5,000 units every 12 hours [14]. This treatment was started within 48 hours of the onset of symptoms and lasted for 10 days (range: six to 14 days). The following 90-day outcomes were reported [14].

▪      Treatment with enoxaparin was associated with a significant reduction in VTE events compared with heparin treatment.

▪      Enoxaparin, however, showed a statistically nonsignificant reduction in PE.

▪      The occurrence of both major extracranial and symptomatic intracranial hemorrhage was not significantly different between both groups.

In addition to the traditional anticoagulants, the novel anticoagulants were also used in prophylaxis. For DVT prophylaxis in recent-onset acute ischemic stroke, the anti-Xa, Danaparoid (ORG 10172), when administered as 1,250 units once daily, is equally safe and efficacious as that of 5,000 IU of heparin sodium [24]. Ximelagatran, a new low-molecular-weight oral prodrug of the direct thrombin inhibitor, has shown precedence over warfarin and this may make it the drug of choice for prevention of VTE.

Other medications have also been evaluated for VTE prophylaxis. In a review of one randomized controlled trial (RCT) with 17,802 participants that assessed rosuvastatin in the primary prevention of VTE, it was seen that when compared with placebo rosuvastatin reduced the incidence of deep vein thrombosis (DVT) (odds ratio (OR) 0.45; 95% confidence interval (CI) 0.25 to 0.79) [25].

Mechanical prophylaxis

Hospitalized patients with acute stroke are at high risk of DVT, and hospitals employ elastic stockings or intermittent pneumatic compression devices alone or in combination for prevention. It was observed in a randomized trial that intermittent pneumatic compression (IPC) combined with elastic stockings seemed to be more effective at reducing the rate of asymptomatic deep vein thrombosis after ICH, as compared to elastic stockings alone (4.7% versus 15.9%) [26]. Proximal DVTs were seen to affect those with below-knee stockings more frequently than those who employed thigh-length stockings [27]. The published data does not support the use of thigh-length graduated compression stockings (GCS) in patients admitted to hospital with acute stroke. The routine use of graduated compression stockings to reduce the risk of DVT after acute stroke is not supported by evidence gathered from randomized trials. As far as the role of IPC use for DVT risk reduction in acute stroke is concerned, the routine use of IPC to reduce the risk of DVT in acute stroke cannot be supported by sufficient evidence, and further randomized studies of IPC are needed to evaluate risks and benefits of this intervention accurately [28]. The decision to use GCS may apply to certain acute stroke patients [29]. Hence, there is insufficient evidence from randomized trials to support the routine use of physical methods for preventing DVT in acute stroke.

Data suggests that old age, female sex, bedridden patients, and high DVT assessment scores >/= 2 are all independent risk factors for DVT in acute stroke patients that require monitoring and prophylaxis. Two-thirds of such cases can be identified on admission with the help of ultrasonography, which is also effective for DVT detection in the rehabilitation setting [27]. Following a stroke, patients may be treated with anticoagulants, mechanically or in a combination of the two. Non-ambulatory stroke patients, in particular, have an increased risk of DVT and PE. When pneumatic sequential compression devices (SCDs) are added to treatment alongside subcutaneous heparin and anti-embolic hose, reduced risk of DVT and PE are observed. This adjunctive treatment with SCD’s should be considered for DVT prophylaxis in nonambulatory stroke patients.

The Clots in Legs Or sTockings after Stroke (CLOTS) 3 trial involved 2,876 stroke patients at 94 hospitals across the United Kingdom, and thigh-length intermittent pneumatic compression (IPC) was used. This particular study was a multicenter parallel-group randomized trial. Patients were enrolled from Day 0 to Day 3 of admission and allocated via a central randomization system (ratio: 1:1) to receive either IPC or no IPC. The CLOTS 3 study showed a 29% reduction in life-threatening DVT and a 14% reduction in overall mortality for patients receiving thigh-length IPC therapy [30].

Data does not support the use of thigh-length GCS in patients admitted to hospital with acute stroke. However, the CLOTS 3 Trial did show a 29% reduction in life-threatening DVT and a 14% reduction in overall mortality for patients receiving thigh-length IPC therapy [30].

DVT prevention in the rehabilitation phase

In stroke patients during the rehabilitation phase, conventional methods for prevention of DVT include adjusted-dose heparin, intermittent pneumatic compression (IPC), and functional electrical stimulation. The incidence of DVT in this phase has also been elevated and was found to occur more as distal DVT on the affected side. The contributing factors in this phase are lower limb paresis, gait disturbance, calf muscle spasticity, and use of ankle-foot orthosis (AFO). A plausible mechanism is that micro-injuries in the venous endothelium may result due to the spasticity and AFO. This damage can be prevented with the help of Cilostazol which seems to be effective following a DVT [31]. Studies evaluating data from Asian neuro-rehabilitation admissions revealed that asymptomatic lower limb DVT is uncommon in that region. These results may be multifactorial and include genetic or ethnic protective factors, early walking initiated rehabilitation, and timing of the admission protocol (median of 14 days post-event) when the maximal thrombotic risk decreases [32]. The low incidence of early DVT in hospitalized stroke patients of Asian ethnicity does not necessitate routine screening in this population. Further research to validate this should ideally include a comparison test for DVT, as ultrasonography may have an inherently lower sensitivity in an asymptomatic population. The incidence of venous thromboembolism is high and greatest in bedridden or wheelchair-bound patients undergoing stroke rehabilitation. Further randomized trials evaluating the safety and efficacy of screening and prophylaxis of DVT in such patients are needed. Thromboprophylaxis for DVT in acute stroke patients in China is inadequate, and there is a higher usage of antiplatelet agents than anticoagulants, which has been shown to be less effective [33].

DVT prophylaxis in intracranial hemorrhage

The role of low-dose heparin treatment in patients with intracranial hemorrhage after 48 hours of onset of symptoms is not associated with an increased hematoma growth and can be used for DVT and PE prophylaxis [34]. A unique algorithm that would reduce the possibility of error in a therapeutic sense should balance various prophylactic strategies for these patients. That is why the use heparin or low-molecular-weight heparin (LMWH) in preventive doses is recommended for patients presenting with acute stroke and limited mobility if there is no contraindication for anticoagulants, along with physical therapy and mechanical methods of prophylaxis [35]. ICH patients who survived the first two days after onset and were subsequently treated with enoxaparin, 20 mg daily, did not show an increased mortality compared to patients not on treatment [36].

In particular, LMWH has been associated with a significantly lower incidence of DVT within 14 days [37]. Both aspirin and mechanical prophylaxis are suboptimal to prevent VTE. Based on studies of large numbers of stroke patients, aspirin leads to a modest reduction, if any, in pulmonary embolism. The American College of Chest Physicians (ACCP) recommends against the use of aspirin alone as a VTE prophylaxis in stroke [38]. Please review Table 1 for the detail of studies on the DVT in acute stroke, Table 2 for the different guidelines, and Table 3 on the cost comparison.

**Table 1:** Studies on Deep Vein Thrombosis in Acute Stroke CDU: compression duplex ultrasound; CRP: C-reactive protein; DVT: deep venous thrombosis; ECH: extracranial hemorrhage; ECS: elastic compression stockings; GCS: graduated compression stockings; GWTG-Stroke: Get With The Guidelines–Stroke; ICH: intracranial hemorrhage; IPC: intermittent pneumatic compression; IU: international units; IVCF: inferior vena cava filters; LMWH: low molecular weight heparin; N/A: not available; PE: pulmonary embolism; RCTs: randomized controlled trials; SC: subcutaneous; SCDs: sequential compression devices; SICH: symptomatic intracranial hemorrhage; UFH: unfractionated heparin; VTE: venous thromboembolism

Study author(s)	Type of study	# of patients	Treatments studied	Primary efficacy endpoints and results	Safety endpoints	Comments
Bembenek J, et al. 2011 [1]	Cohort prospective study	299	LMWH as given to the patients with the high risk of DVT.	N/A	N/A	Additional care to patients with increased serum CRP levels.
Soroceanu A, et al. 2016 [2]	Retrospective review	448	Patients undergoing spinal surgery.	Medical complications including stroke, DVT, and PE, were studied.	N/A	N/A
Kamran SI, et al. 1998 [3]	Clinical trial	233 Grp A, 432 Grp B, 16 Grp C	Pneumatic SCDs, subcutaneous heparin, and anti-embolic hose	N/A	N/A	Adding SCDs to treatment with subcutaneous heparin and anti-embolic hose reduced the risk of DVT and PE.
Kelly J, et al. 2001 [7]	Review Article	N/A	IVCF, Anticoagulants	N/A	N/A	Early use of short-term, low-dose, UFH is not associated with sustained, clinically meaningful benefit
Dennis M, et al. 2011 [15] CLOTS (Clots in Legs Or sTockings after Stroke) Trial Collaboration	Randomized trial	(3,114 Total) 1,552 with thigh-length stockings 1,562 with below-knee stockings	Thigh length vs. below knee stockings	Proximal DVT, alive and free of the primary outcome, or died before any primary outcome.	Dead by 30 days; symptomatic or asymptomatic proximal DVT; any symptomatic or asymptomatic DVT affecting the calf, popliteal, or femoral veins; or pulmonary emboli within 30 days.	Unfortunately, models based on clinical factors alone discriminate poorly between immobile patients with stroke at high and low risk, and would not facilitate individual tailoring of DVT prophylaxis strategies.
Kamerkar DR, et al. 2016 [16]	Retrospective review	549	Confirmed diagnosis of VTE. DVT confirmed by Doppler ultrasonography.	N/A	N/A	Bleeding was not the limiting factor for anticoagulant treatment in most patients.
Paciaroni M, et al. 2011 [18]	Review article	1,000 (4 studies)	Comparing anticoagulants with other treatments like elastic stockings and IPC.	Symptomatic and asymptomatic DVT, symptomatic and asymptomatic pulmonary embolisms, and death at the final time of follow-up (varying between seven days and three months)	Symptomatic and asymptomatic hematoma enlargement	Early anticoagulation is associated with a significant reduction in PE, no substantial reduction in death, and a non-significant increase in hematoma enlargement.
Bath PM, et al. 2000 [19]	Systemic review of RCTs	3,048 (11 completed RCTs)	LMWH	N/A	N/A	LMWHs do reduce the risk of DVT and PE but only at the expense of an increased risk of major extracranial hemorrhage and probably SICH.
Kamphuisen PW, et al. 2005 [21]	Review article	(Multiple studies)	Mechanical methods, anticoagulants.	N/A	N/A	Higher doses increase the risk of cerebral bleeding and should be avoided for prophylactic use. Both aspirin and mechanical prophylaxis are suboptimal to prevent VTE. GCS should be reserved for patients with a clear contraindication to antithrombotic agents.
Kamphuisen PW, et al. 2005 [22]	Review article	23,043 (16 trials)	DVT prophylaxis	N/A	N/A	Low-dose LMWH had the best benefit/risk ratio in patients with acute ischemic stroke by decreasing the risk of both DVT and pulmonary embolism, without a clear increase in ICH or ECH.
Dumas R, et al. 1994 [24]	Clinical trial	179	Org 10172 (1250 anti-Xa units SC once daily); heparin sodium (5,000 IU SC twice daily)	N/A	N/A	1,250 anti-Xa units of Org 10172 once daily was both safe and as effective as 5,000 IU of heparin sodium twice daily for DVT prophylaxis in patients with acute ischemic stroke of recent onset.
Naccarato M, et al. 2010 [28]	Review Article	2,615 (2 RCTs of GCS); 177 (2 studies of IPC)	GCS, IPC, ECS	Events during scheduled treatment period: 1) Deaths from any cause; 2) DVT; 3) Fatal or non-fatal PE. Adverse effects	Events during scheduled follow-up period: 1) Deaths from any cause; 2) DVT; 3) Fatal or non-fatal PE.	Did not support the routine use of GCS. Insufficient evidence to support IPC.
Xu B, et al. 2010 [29]	Review article	Muir: 65; CLOTS1: 2,518; Cochrane: 123	GCS	N/A	N/A	GCS increased the risk of skin problems in this population. They may also increase the risk of critical limb ischemia and are contraindicated in patients with the known peripheral vascular disease or an ankle-brachial index <0.8.
Dennis M, et al. 2013 [30] CLOTS (Clots in Legs Or sTockings after Stroke) Trial Collaboration	Randomized trial	5,632	Efficacy and safety of GCS	The occurrence of asymptomatic or an asymptomatic DVT in the popliteal or femoral veins detected by CDU or asymptomatic DVT in the popliteal or femoral veins, which had been confirmed on imaging, within 30 days of randomization.	Secondary outcomes relevant to this analysis include death, and `any DVT’ (including the calf, popliteal or femoral) and `symptomatic DVT` within 30 days.	Models based on clinical factors alone discriminate poorly between immobile patients with stroke at high and low risk.
Hara Y, et al. 2008 [31]	Original study	272	Antiplatelet (Cilostazol) and anticoagulants	N/A	N/A	Cilostazol seemed effective in protecting again venous endothelial damage following DVT.
Chua K, et al. 2008 [32]	Prospective observational single-center	419	Mechanical prevention. Anticoagulants (in selected population).	N/A	N/A	Asymptomatic lower limb DVT is indeed uncommon in Asian neurorehabilitation admissions.
Orken DN, et al. 2009 [34]	Prospective randomized study	75	LMWH and GCS	Development of symptomatic or asymptomatic DVT or PE.	Enlargement of hemorrhage. The occurrence of new hemorrhage.	Low-dose heparin treatment after 48 hours of stroke in ICH patients is not associated with an increased hematoma growth and should be used for DVT and PE prophylaxis.
Zubkov AY, et al. 2009 [35]	Review article	(Multiple RCTs)	Mechanical prevention, anticoagulants	N/A	N/A	Mechanical devices, such as IPCs, significantly decrease the occurrence of asymptomatic DVT for patients with ICH as compared with elastic stockings alone, although this advantage was not found in a meta-analysis of prospective studies
Tetri S, et al. 2008 [36]	Retrospective study	407	Enoxaparin (LMWH)	N/A	Hematoma enlargement	No increased mortality among ICH patients who survived the first two days after the onset of ICH and were afterward treated with enoxaparin.
Bravata DM, et al. 2010 [39]	Retrospective cohort study	1,487	Deep vein thrombosis (DVT) prophylaxis, and early mobilization.	Combined endpoint of hospital mortality, discharge to hospice, or discharge to a skilled nursing facility.	N/A	Patients with stroke who received a DVT prophylaxis were less likely to have poor outcomes.
Smith EE, et al. 2009 [40]	Registry	479, 284 (Consecutive stroke and TIA admissions.)	Acceptable treatments: Pneumatic compression devices and anticoagulants	N/A	N/A	GWTG-Stroke database participation was associated with improving quality of care for hemorrhagic stroke
Dennis M, et al. 2015 [41] CLOTS (Clots in Legs Or sTockings after Stroke) Trial Collaboration	Randomized trial	2,876	Thigh-length sleeves to both legs	The occurrence of a symptomatic or asymptomatic proximal DVT confirmed on CDU within 30 days of randomization.	Survival to six months; disability; and hospital costs (based on the cost of IPC and length of hospital stay).	IPC appeared to reduce the risk of DVT and probably improved survival in all immobile stroke patients. IPC should be considered in all immobile stroke patients, but that the final decision should be based on individual’s prognosis.
Hadziahmetovic NV, et al. 2009 [42]	Original study	86	Aspirin, Physical therapy.	N/A	N/A	For patients with acute stroke and limited mobility, it was recommended to use heparin or LMWH in preventive doses if there are no contraindications for anticoagulants, with physical therapy and mechanical methods of prophylaxis.
Zheng H, et al. 2008 [43]	Multicenter prospective cohort study	656	Antiplatelets, anticoagulants, IPC, and stockings	N/A	N/A	Guidelines for preventing DVT in acute stroke should be established, and efforts should be made to improve venous thromboembolism prophylaxis practice
Tan SS, et al. 2007 [44]	Case reports	44	N/A	N/A	N/A	The institution of early DVT screening with Doppler ultrasound for stroke patients was not recommended.
Rabadi MH, et al. 2009 [45]	Review article	(35 RCTs)	Compression stockings, IPC	N/A	N/A	Exercise programs for community-dwelling stroke patient helped maintain and even improve their functional state.
Hills NK, et al. 2006 [46]	Cohort registry	16,301	DVT prophylaxis	N/A	N/A	Three targeted quality-improvement measures improved among hospitals participating in a disease-specific registry.
Zorowitz RD, et al. 2005 [47]	Cohort registry	1161	Warfarin, heparin, enoxaparin, dalteparin, and alteplase	N/A	N/A	Unless patients have any medical contraindications to these medications, they should receive these evidence-based treatments for secondary stroke prophylaxis.
Roderick P, et al. 2005 [48]	Review article	(Multiple RCTs)	Mechanical methods, oral anticoagulation, dextran, and regional anesthesia as thromboprophylaxis.	DVT, PE, and major bleeding events	Proximal venous thrombosis (PVT) and fatal PE	There was little information on the prevention of VTE among high-risk medical patients (such as those with stroke), so further randomized trials in this area would be helpful.
Wilson RD, et al. 2005 [49]	Prospective study	N/A	(Cost-effectiveness analysis)	N/A	N/A	This study estimates that the cost-effectiveness ratio was considerably higher than that reported in other rehabilitation conditions.
Jaff MR, et al. 2005 [50]	Multicenter prospective cohort study	5,451	IVCF placement	N/A	N/A	Improved physician education regarding mechanical and pharmacologic prophylaxis alternatives might reduce the use of IVCFs.

**Table 2:** Different Guidelines on the Management of DVT After an Acute Stroke. ASA: acetyl salicylic acid; CDU: compression duplex ultrasound; CRP: C-reactive protein; DVT: deep venous thrombosis; ECH: extracranial hemorrhage; ECS: elastic compression stockings; GCS: graduated compression stockings; GWTG-Stroke: Get With The Guidelines–Stroke; ICH: intracranial hemorrhage; IPC: intermittent pneumatic compression; IU: international units; IVCF: inferior vena cava filters; LMWH: low molecular weight heparin; NICE: The National Institute for Health and Care Excellence; PE: pulmonary embolism; SC: subcutaneous; SCDs: sequential compression devices; UFH: unfractionated heparin; VTE: venous thromboembolism

	Ischemic stroke	ICH
US Guidelines	Grade 1A: Pts. with restricted mobility, prophylactic low-dose SC heparin or LMWH. Grade 1B: Pts. Contraindications to anticoagulants use IPC devices or elastic stockings.	Grade 1 B: Pts. with an acute ICH, the initial use of IPC devices is recommended. Grade 2 C: In stable patients, use low-dose SC heparin as soon as the second day after the onset of hemorrhage.
Canadian Guidelines	1. Early mobilization and adequate hydration should be encouraged for all acute stroke patients to help prevent VTE (Evidence level C) 2. Patients at high risk of VTE should be started on VTE prophylaxis immediately (Evidence level A). a. LMWH should be considered for patients with acute ischemic stroke at high risk of VTE, or UFH for patients with renal failure (Evidence level B). b. The use of anti-embolism stockings alone for post-stroke VTE prophylaxis is not recommended (Evidence level A).	3. There is insufficient evidence on the safety and efficacy of anticoagulant DVT prophylaxis after ICH (Evidence level C). Antithrombotic and anticoagulants should be avoided for at least 48 hours after onset (Evidence level C).
British Guidelines	Heparin/LMWH for prevention of venous thromboembolism after stroke only when situations of high-risk of DVT and PE arise, such as patients with major restriction of mobility, previous history of VTE, dehydration or comorbidities (such as malignant disease), and there is a low risk of bleeding.	Treatment to prevent the development of further pulmonary emboli using either anticoagulation or IVCF. (NICE guidelines)
Italian Guidelines	GCS and IPC should not be used as the only prophylactic strategy (Grade B). Use of GCS as the only prophylactic strategy in patients with contraindications to pharmacological prophylaxis (Grade B). IPC should be applied in combination with GCS in patients with contraindications to pharmacological prophylaxis (Grade B). We recommend the routine use of prophylactic doses of either LMWH or UFH (5,000 IU t.i.d) for the prevention of VTE in patients with acute ischemic stroke (Grade A). LMWH should be preferred over UFH (Grade B). Treatment should be started within 48 hours of the acute event and should continue for approximately 14 days (Grade A). Treatment should not be administered to patients with evidence of hemorrhagic transformation (Grade D). The use of pharmacological prophylaxis should not be a contraindication for the concomitant administration of ASA (Grade B). ASA is not recommended for the prevention of DVT and PE in patients with acute ischemic stroke (Grade A).	GCS in patients with concomitant immobilization (Grade D). The need to combine the use of GCS with IPC is uncertain (Grade D). We also suggest considering the use of LMWH in immobilized patients. Patients defined at particularly high risk for VTE (Grade D). The benefit of UFH as an alternative to LMWH is uncertain (Grade D). We suggest not using ASA for the prevention of VTE (Grade D).
Australian Guidelines	a) Early mobilization and adequate hydration should be encouraged with all acute stroke patients to help prevent DVT and PE. b) Antiplatelet therapy should be used for people with ischaemic stroke to prevent DVT/PE. (Level I) c) The following interventions may be used with caution for selected people with acute ischaemic stroke at high risk of DVT/PE: • LMWH or heparin in prophylactic doses; Level I and Level II. • Thigh-length antithrombotic stockings. Level II	

**Table 3:** Cost Comparison of Different Medications Used in the Management of DVT. LMWH: low molecular weight heparin; UFH: unfractionated heparin

Medication	Cost
Heparin, UFH/LMWH	$188/day
Enoxaparin	$131.96/day
Warfarin	$0.46/day
Dabigatran	$4.09/day
Fondaparinux	$59.3/day

## Conclusions

In conclusion, in light of the literature review discussed in this manuscript, we recommend the following:

For ischemic stroke, we recommend low-dose anticoagulant therapy, 5,000 units subcutaneously every eight hours.

For intracerebral hemorrhage, place SCDs on admission. On Day 2, if the patient is stable, initiate unfractionated heparin, 5,000 units subcutaneously every eight hours. On Days 10-14, if the patient is stable, one may switch to chronic oral anticoagulant therapy if there is a high risk (> 7%/yr.) for cardioembolic stroke, with prior deep ICH at low risk (< 1.4%/yr.) for recurrence. On discharge to a facility: continue until ambulatory.

In a subarachnoid hemorrhage, on admission place SCDs. On Day 2, if stable, initiate unfractionated heparin, 5,000 units subcutaneously every eight hours.
